# Quantitative structure–activity relationship to predict the anti-malarial activity in a set of new imidazolopiperazines based on artificial neural networks

**DOI:** 10.1186/s12936-019-2941-5

**Published:** 2019-09-14

**Authors:** Saeed Yousefinejad, Marjan Mahboubifar, Rayhaneh Eskandari

**Affiliations:** 1 Research Center for Health Sciences, Institute of Health, Department of Occupational Health Engineering, School of Health, Shiraz University of Medical Sciences, Shiraz, Iran; 2 Medicinal and Natural Products Chemistry Research Center, Shiraz University of Medical Sciences, Shiraz, Iran; 3 Department of Chemistry, Shiraz Branch, Islamic Azad University, Shiraz, Iran

**Keywords:** Antimalarial, Imidazolopiperazine, QSAR, Artificial neural networks

## Abstract

**Background:**

After years of efforts on the control of malaria, it remains as a most deadly infectious disease. A major problem for the available anti-malarial drugs is the occurrence of drug resistance in *Plasmodium*. Developing of new compounds or modification of existing anti-malarial drugs is an effective approach to face this challenge. Quantitative structure activity relationship (QSAR) modelling plays an important role in design and modification of anti-malarial compounds by estimation of the activity of the compounds.

**Methods:**

In this research, the QSAR study was done on anti-malarial activity of 33 imidazolopiperazine compounds based on artificial neural networks (ANN). The structural descriptors of imidazolopiperazine molecules was used as the independents variables and their activity against 3D7 and W2 strains was used as the dependent variables. During modelling process, 70% of compound was used as the training and two 15% of imidazolopiperazines were used as the validation and external test sets. In this work, stepwise multiple linear regression was applied as the valuable selection and ANN with Levenberg–Marquardt algorithm was utilized as an efficient non-linear approach to correlate between structural information of molecules and their anti-malarial activity.

**Results:**

The sufficiency of the suggested method to estimate the anti-malarial activity of imidazolopiperazine compounds at two 3D7 and W2 strains was demonstrated using statistical parameters, such as correlation coefficient (R^2^), mean square error (MSE). For instance R^2^_train_ = 0.947, R^2^_val_ = 0.959, R^2^_test_ = 0.920 shows the potential of the suggested model for the prediction of 3D7 activity. Different statistical approaches such as and applicability domain (AD) and y-scrambling was also showed the validity of models.

**Conclusion:**

QSAR can be an efficient way to virtual screening the molecules to design more efficient compounds with activity against malaria (3D7 and W2 strains). Imidazolopiperazines can be good candidates and change in the structure and functional groups can be done intelligently using QSAR approach to rich more efficient compounds with decreasing trial–error runs during synthesis.

## Background

After years of efforts to fight and control of malaria, it is still a prevalent and deadly infectious disease, especially in the third-world countries in Africa, Asia, and South America [1, 2]. The estimated deaths because of malaria in 2015 were 429,000 (range 235,000–639,000), which were mainly distributed in the Africa (92%), Southwest Asia (6%) and the Eastern Mediterranean (2%) [3]. The pregnant women and children below 5 years of age are the more vulnerable groups, and about 85% of deaths occurring in children with this age range [4].

The disease is caused by a parasite of the genus *Plasmodium*. The main species of *Plasmodium* are *Plasmodium falciparum*, *Plasmodium vivax*, *Plasmodium ovale, Plasmodium knowlesi* and *Plasmodium malariae,* with *P. falciparum* responsible for most of the mortality [1, 4].

Many compounds with anti-malarial activity have been described, including quinine, chloroquine, proguanil, pyrimethamine, artemisinin, mefloquine, atovaquone [5]. The major problem in the treatment of malaria is that *Plasmodium* parasites become resistant to anti-malarial drugs. The most commonly used anti-malarial drug, chloroquine, became ineffective due to rapidly spreading resistance of *P. falciparum* to this compound; the newer anti-malarial drugs, such as mefloquine or artemisinins also face to resistance problem. The other problem in control of malaria is the lack of an effective vaccine for this disease. Therefore, developing new anti-malarial agents is a necessity and chemical modification of existing compounds is one of the strategies available [1].

In silico methods, such as quantitative structure activity relationship (QSAR), molecular docking and pharmacophore modelling by decreasing the time and cost of drug discovery play a significant role in the field of drug design and development [6]. QSAR can provide a mathematical relationship of the physicochemical properties and structural features that is required for a specific activity for a set of similar compounds. In this way, synthesis of potential candidate molecules can be performed by focusing on the chemical characteristics which have influenced on a specific activity [7].

QSAR methods have previously been used to investigate anti-malarial compounds. In 2001, Agrawal et al. studied the anti-malarial activity of a series of sulfonamide derivatives (2,4-diamino-6-quinazoline sulfonamides) [8]. In 2002, 3D-QSAR studies on the artemisinin analogues were performed by Cheng et al., a study done on the basis of the docking models employing comparative molecular force fields analysis (CoMFA) and comparative molecular similarity indices analysis (CoMSIA) [9]. Katritzky et al. investigated two various set of compounds for each of two strains D6 and NF54 of *Plasmodium falciparum* using QSAR modelling with CODESSA PRO software in 2006 [10]. A study on anti-malarial artemisinin derivatives was done by Cardoso et al. in 2008 using molecular electrostatic potential (MEP) maps and multivariate QSAR [11]. In 2015, Ojha and Roy reported the status of anti-malarial drug research from the year 2011 to 2014 with special reference to application of QSAR models. In their report, aminoquinolines as a group of anti-malarial compounds were analysed by various research groups using QSAR models; the other groups of compounds were endochin analogs, artemisinin analogs, aurone chalcone, prodiginines, acridine, hydroxypyridinones and cycloguanil derivatives, which their QSAR modelling reported [7]. In 2018, Cheoymang and Na-Bangchang in a systematic review article reported about application of in silico models for anti-malarial drug discovery in the years between 2008 and 2015. In this article 2D- or 3D-QSAR is mentioned as one of the commonly applied in silico methods for investigating on anti-malarial compounds [12].

Imidazolopiperazine is a class of anti-malarial compounds, including KAF156 (also known as GNF156) which is active against a wide range of *Plasmodium* species and in phase 2 trials have shown better or analogous parasite killing rates compared to the effective artemisinin-based combination therapy (ACT) [13, 14]. In this article, the anti-malarial activity of a set of imidazolopiperazines was investigated using quantitative structure activity relationship. Artificial neural networks were used for modelling the activity of 33 imidazolopiperazines derivatives.

## Methods

### Data set

A data set consisting of imidazolopiperazines reported by Wu et al. and Nagle et al. [15, 16] was used for this study. A set of 33 compounds was selected which their structural skeleton and the name of compounds are displayed in Table 1.

**Table 1:**
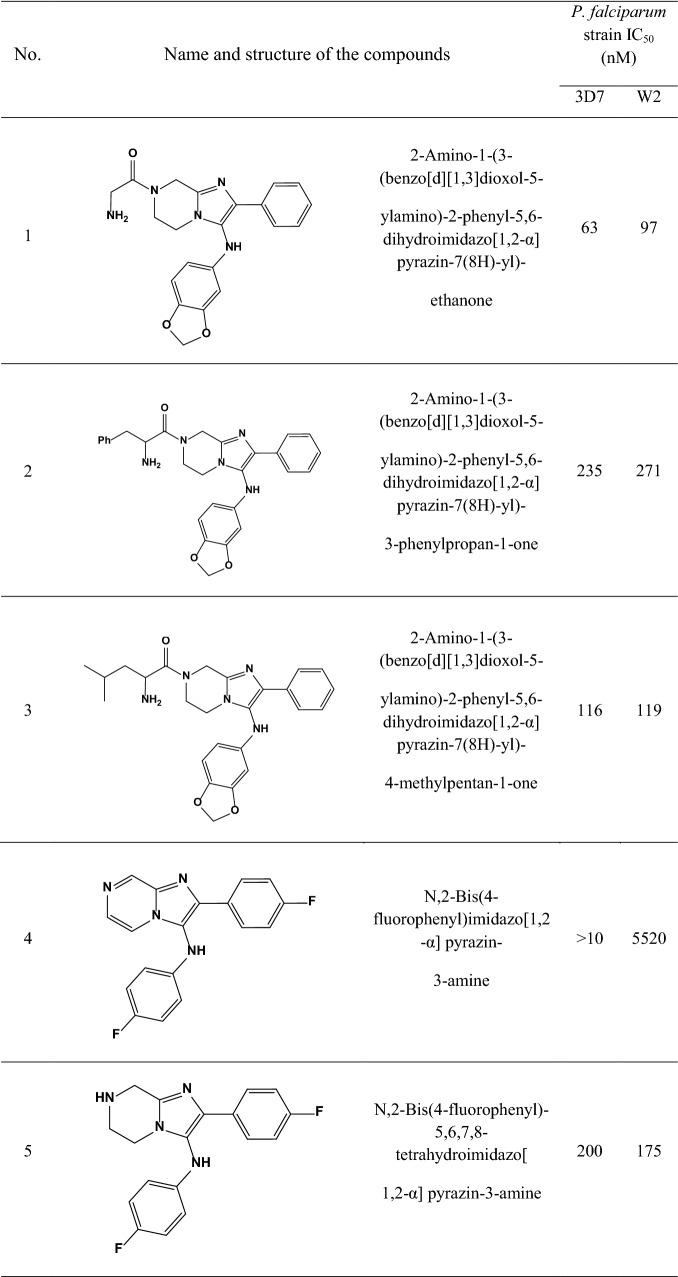

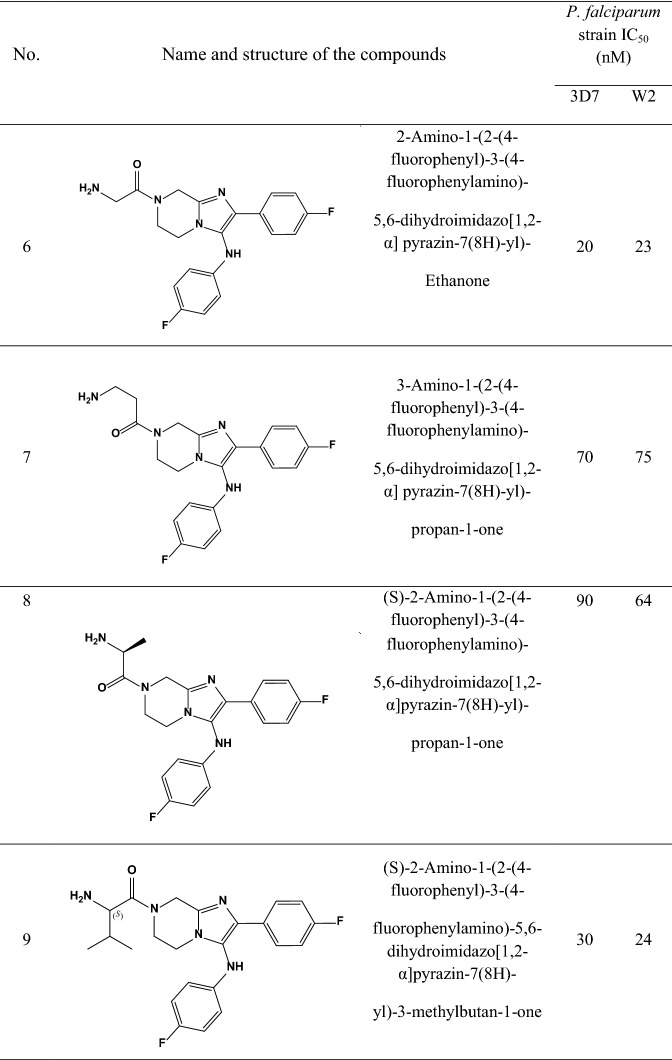

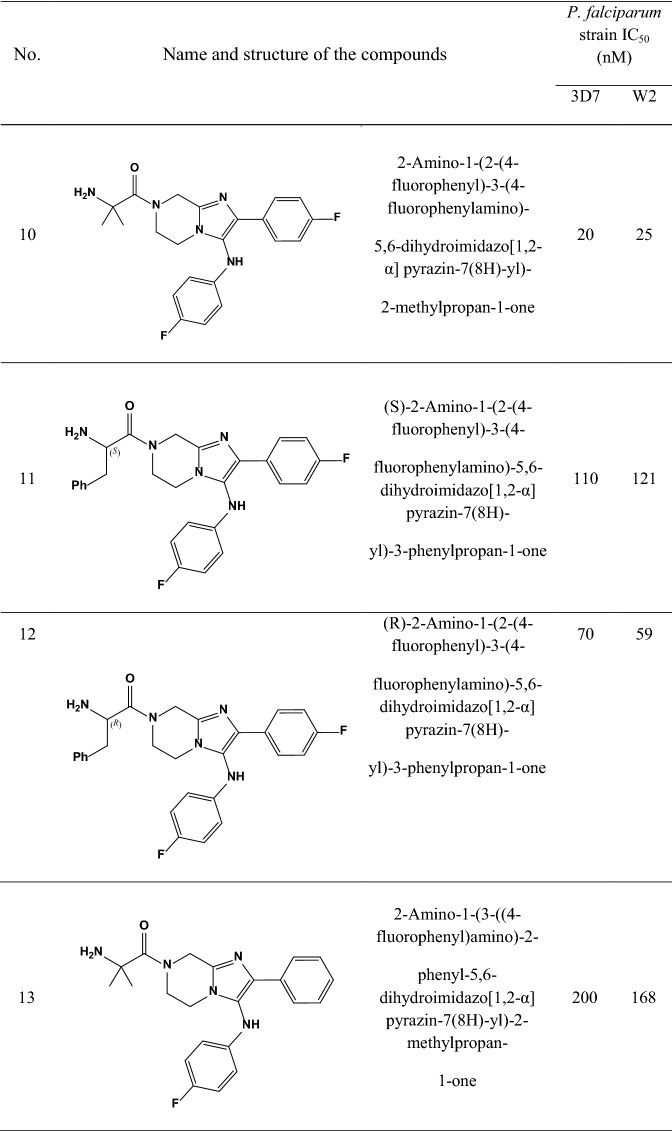

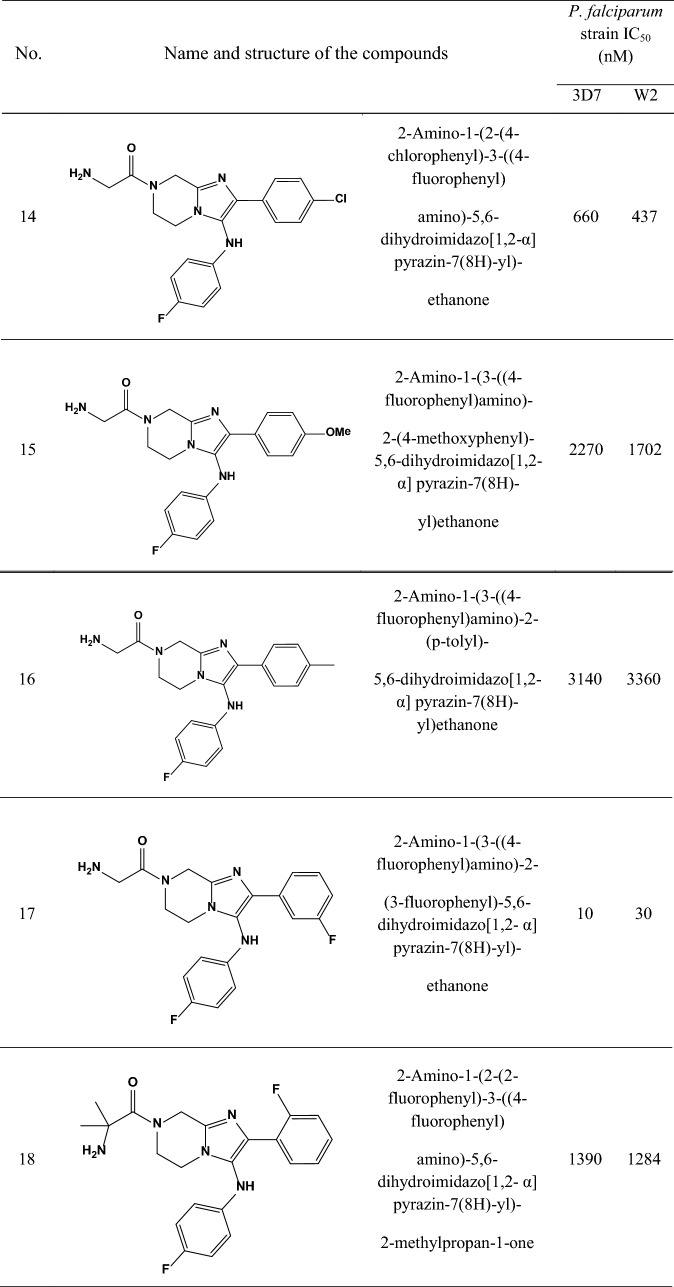

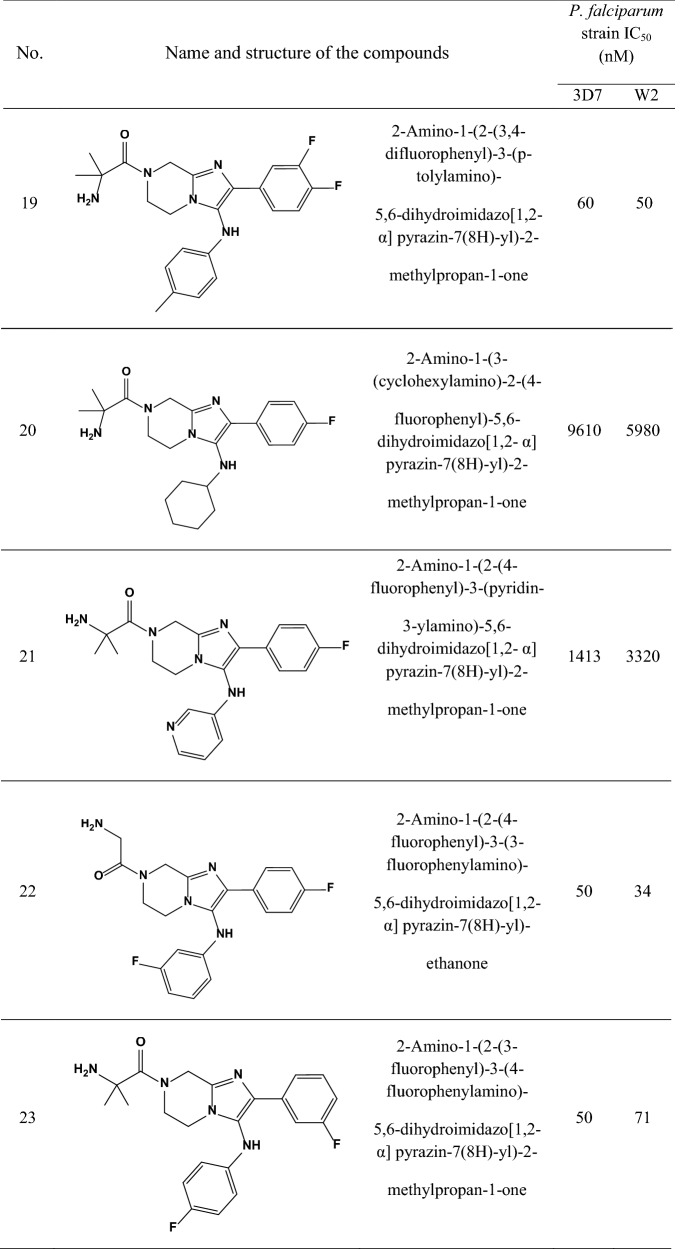

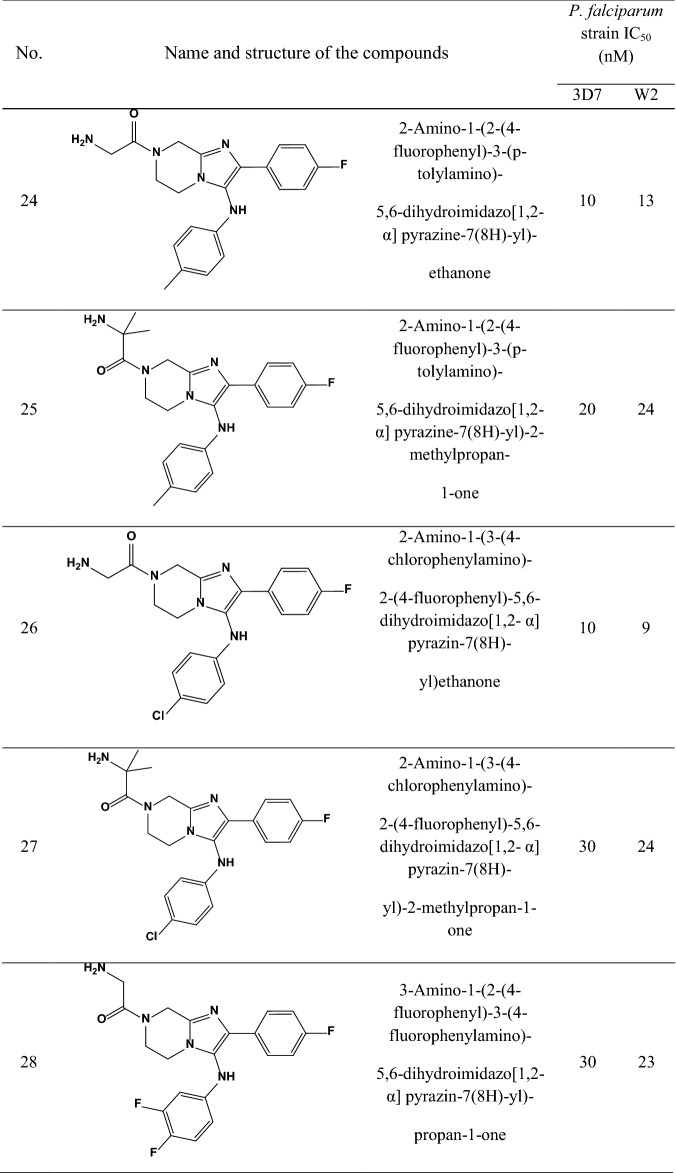

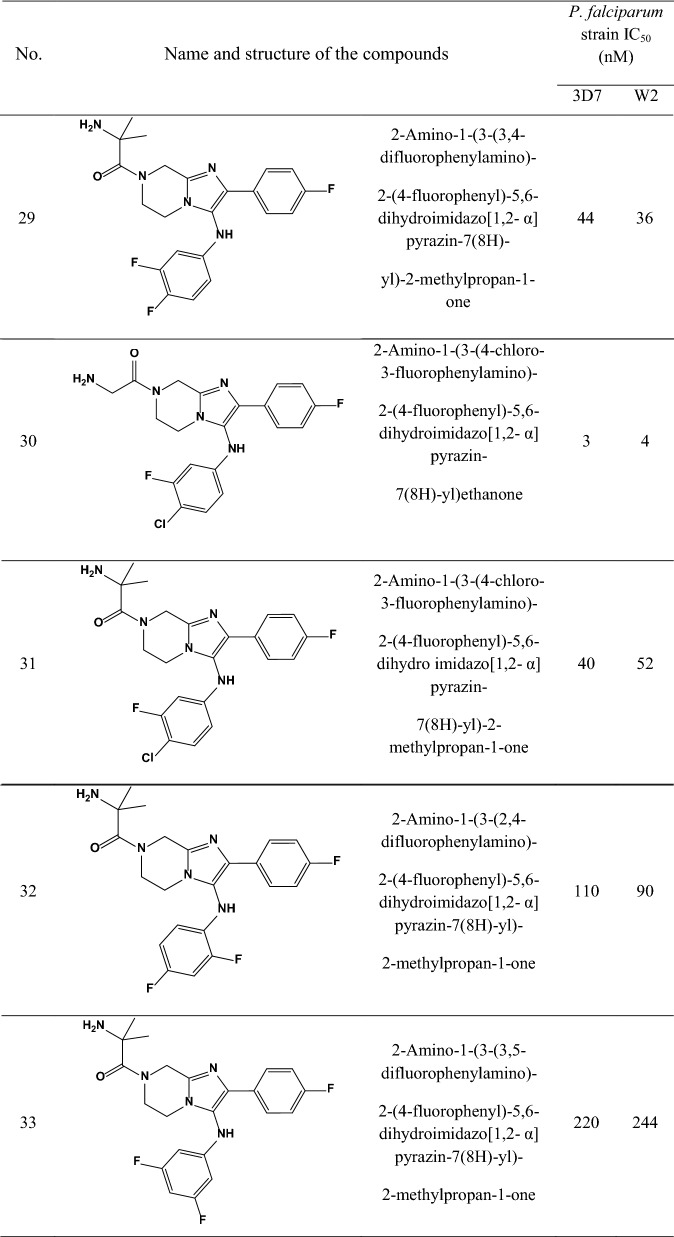
Structures of imidazolopiperazine derivatives and their biological activities (IC50, nM) for 3D7 and W2

### Descriptor generation and pretreatment

After drawing the 2D structures of the 33 imidazolopiperazine derivatives using HyperChem software (Ver. 8.0.3, Hypercube Inc., Gainesville, USA), the geometries of the molecules were fully optimized using the semi-empirical AM1 method. The optimization was done until the root mean square gradient achieves 0.001 kcal mol^−1^ or 1000 cycles for all the molecular structures.

The resulting optimized geometries were transferred to the DRAGON software [17, 18], and the descriptors were calculated. Then, the same descriptors for all the structures were kept and others were removed. At the first step for pretreatment of the descriptors, the constant or near-constant variables among the remained descriptors were removed. At the second step, for decreasing the redundancy existing in the descriptors, the correlation of descriptors with each other and with the biological activities (pIC_50_) against 3D7 and W2 was examined and among the collinear ones (r > 0. 95), the descriptors that had the highest correlation with pIC_50_ for 3D7 and W2 were retained. After these steps, the number of remaining descriptors for all the 33 compounds in each mode (against 3D7 and W2) was about 555 which were collected in an n × m data matrix (D), where n and m are the number of imidazolopiperazine derivatives (= 33) and the number of descriptors (= 555), respectively. The data set was randomly divided to training set with 23 compounds, test and validation set, each of them include 5 compounds.

It should be noted that the variable selection was done by stepwise multiple linear regression (SMLR) on the training set using SPSS (version 19.0, SPSS Inc., http://www.spss.com). Artificial neural networks were done using MATLAB (version 7.6, Math work, Inc., http://www.mathworks.com). All other statistical calculations and evaluations were also conducted in MATLAB. In ANN modeling, a two-layer feed-forward network with sigmoid hidden neurons and linear output neurons was used with only two hidden neurons. The mean square error was also used as the performance criteria of the network.

## Results

In the first step, due to the preference of using the linear models to the non-linear ones [19], the QSAR modelling of the mentioned imidazolopiperazine derivatives with anti-malarial activities was investigated using the linear models. This effort did not have good results, by using multiple linear regression (MLR) and partial least square (PLS) models, and forced the authors to test the nonlinear models.

**Fig. 1:**
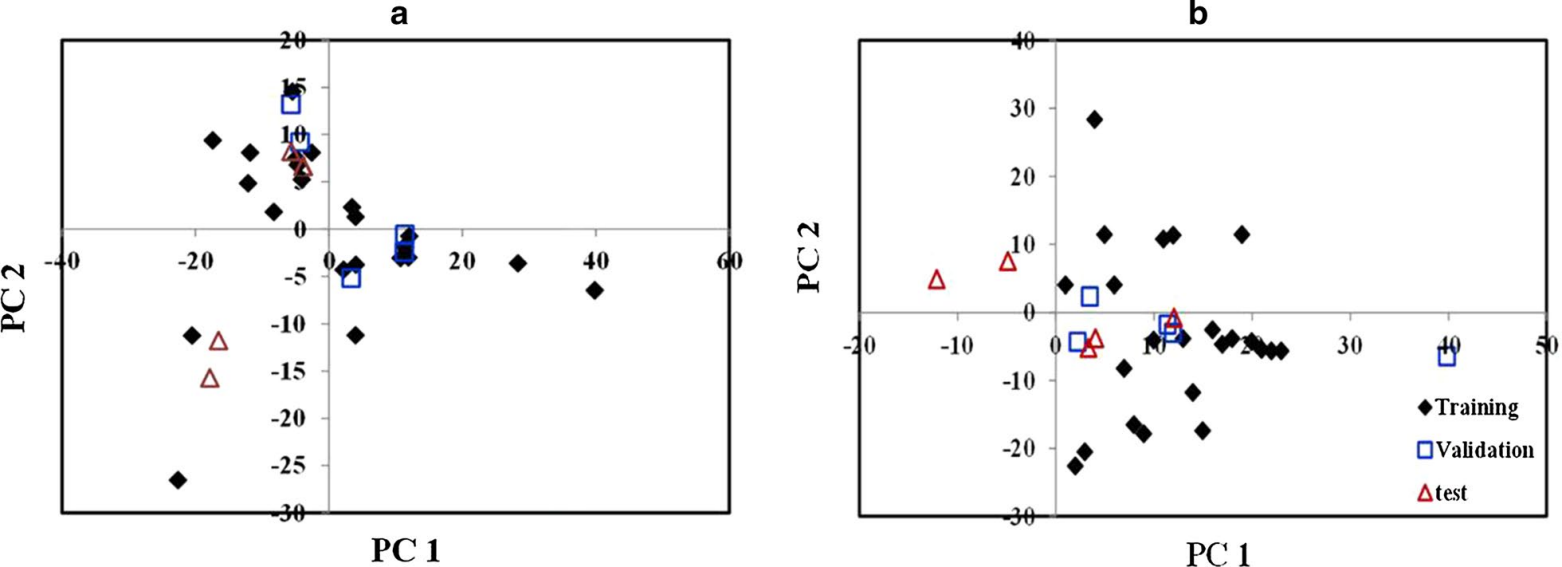
Random distribution of the training, validation, and test sets at two-dimensional PCA plot (PC1 vs. PC2) related to **a** 3D7 and **b** W2

At the next step for evaluation the randomized distribution of the molecules belong to the three data set (the training, validation and test sets) in the space of descriptors, principal component analysis (PCA) was applied. The two-dimensional (2D) PCA plot (PC1 vs. PC2) of imidazolopiperazine derivative molecules for the data of two models (3D7 and W2) is displayed in Fig. 1.

### Variable selection

Variable selection was done using SMLR on the 23 × 555 data matrix. The statistic parameters like Fisherʼs F value (F) and correlation coefficient (R^2^) were employed for evaluation the goodness of the selected variables and as fitting criteria. In this way, variables with the most significant values of F and highest correlation coefficient were selected by inserting into/removing from the model respectively and 12 variables were selected by using this approach. In the next step, the models with 1 to 12 variables were checked using ANN method for training and validation sets [20] and it was found that in the models with more than 6 variables despite of improvement in the training set results, the prediction ability of the validation set reduced because of overfitting [21]. The results of SMLR for the selected variables are summarized in Additional file 1: Tables S1–S3 for the 3D7 model and in Additional file 1: Table S4–S6 for the W2 model.

So the number of 6 variables was selected for both 3D7 and W2 models. The 6 selected descriptors for modelling the biological activities (pIC_50_) against 3D7 and W2 are represented in Additional file 1: Tables S7, S8, respectively. It should be mentioned that the results of test set were not considered during selection of the optimum model. The definition of the used molecular descriptors for modelling the biological activities (pIC_50_) for the 3D7 and W2 strain are presented in Table 2.

**Table 2:** The definition of the used molecular descriptors for modelling of two kinds of activities (3D7 and W2)

Molecular descriptors	Definition	Descriptor category	Strain
GATS4m	Geary autocorrelation of lag 4 weighted by mass	2D autocorrelations	3D7
GATS7m	Geary autocorrelation of lag 7 weighted by mass	2D autocorrelations	3D7
Mor06u	Signal 06/unweighted	3D-MoRSE descriptors	3D7
Mor31u	3D-MoRSE, signal 31/unweighted	3D-MoRSE descriptors	3D7
+R3e	R maximal autocorrelation of lag 3/weighted by Sanderson electronegativity	GETAWAY descriptors	3D7
+R2p	R maximal autocorrelation of lag 2/weighted by polarizability	GETAWAY descriptors	3D7
BEHm3	Highest eigenvalue n.3 of Burden matrix/weighted by atomic masses	Burden eigenvalues	W2
MATS7m	Moran autocorrelation of lag 7 weighted by mass	2D autocorrelations	W2
RDF020m	Radial distribution function-020/weighted by mass	RDF descriptors	W2
Mor23u	3D-MoRSE signal 23/unweighted	3D-MoRSE descriptors	W2
Mor20p	3D-Morse signal 23/weighted by polarizability	3D-MoRSE descriptors	W2
MLOGP	Moriguchi octanol–water partition coefficient	Molecular properties	W2

After the selection of the descriptors, the evaluation of correlation was done using the pair-correlation matrix for 23 training compounds and the total of training and test sets (28 compounds). The related data are shown in Tables 3 and 4 for six descriptors of the 3D7 and W2 models respectively.

**Table 3:** The pair correlation coefficient (R^2^) and the variance inflation factor (VIF) for the 6 descriptors at the training and total set for 3D7 model

	GATS7m	Mor31u	Mor06u	R2p+	GATS4m	R3e+	VIF
***GATS7***							
Trainng set	1.00						2.21
Total set^a^	1.00						2.85
***Mor31u***							
Trainng set	0.38	1.00					2.86
Total set^a^	0.37	1.00					1.43
***Mor06u***							
Trainng set	0.00	0.15	1.00				1.44
Total set^a^	0.01	0.12	1.00				1.44
***R2p+***							
Trainng set	0.1	0.25	0.11	1.00			1.47
Total set^a^	0.08	0.21	0.12	1.00			1.46
***GATS4m***							
Trainng set	0.03	0.19	0.12	0.18	1.00		1.49
Total set^a^	0.05	0.26	0.10	0.19	1.00		2.57
***R3e+***							
Trainng set	0.03	0.06	0.00	0.01	0.10	1.00	1.44
Total set^a^	0.11	0.02	0.04	0.01	0.06	1.00	1.52

^a^Total set: total of training, validation and test sets

**Table 4:** The pair correlation coefficient (R^2^) and the variance inflation factor (VIF) for the 6 descriptors at the training and total set for W2 model

	Mor20p	MATS7m	RDF020m	MLOGP	BEHm3	Mor23u	VIF
Mor20p							
Training set	1.00						1.63
Total set^a^	1.00						2.67
MATS7m							
Training set	0.00	1.00					1.62
Total set^a^	0.00	1.00					1.64
RDF020m							
Training	0.02	0.01	1.00				1.56
Total set^a^	0.011	0.00	1.00				1.41
MLOGP							
Training set	0.15	0.18	0.07	1.00			1.91
Total set^a^	0.04	0.20	0.01	1.00			1.55
BEHm3							
Training set	0.01	0.08	0.19	0.00	1.00		1.34
Total set^a^	0.38	0.00	0.01	0.14	1.00		2.24
Mor23u							
Training set	0.14	0.13	0.00	0.00	0.01	1.00	1.38
Total set^a^	0.39	0.19	0.07	0.00	0.15	1.00	2.21

^a^Total set: total of training, validation and test sets

### Model development

At the model development and validation steps, the training set with 23 compounds (70% of the imidazolopiperazine derivative molecules) was used for artificial neural networks modelling. Feed forward artificial neural networks with Levenberg–Marquardt algorithm were used for this purpose. The validation and test sets (each of them with 5 compounds containing 15% of the imidazolopiperazine derivative molecules) were used to validate the prediction ability of the proposed anti-malarial models. The statistical parameters of the used ANN models is represented in Table 5.

**Table 5:** Statistical parameters of the artificial neural networks models used for prediction of anti-malarial activity at 3D7 and W2

	Number of compounds	3D7^a,b^			W2^c,d^		
		R	R^2^	MSE	R	R^2^	MSE
Training set	23	0.973	0.947	0.036	0.964	0.929	0.030
Validation set	5	0.979	0.959	0.051	0.892	0.797	0.290
Test set	5	0.959	0.920	0.254	0.901	0.813	0.740

^a^Average absolute deviation (AAD) for 3D7 model = 0.168

^b^Percentage average absolute relative error (AARE%) for 3D7 model = 2.98%

^c^Average absolute deviation (AAD) for W2 model = 0.257

^d^Percentage average absolute relative error (AARE%) for W2 model = 4.20%

**Fig. 2:**
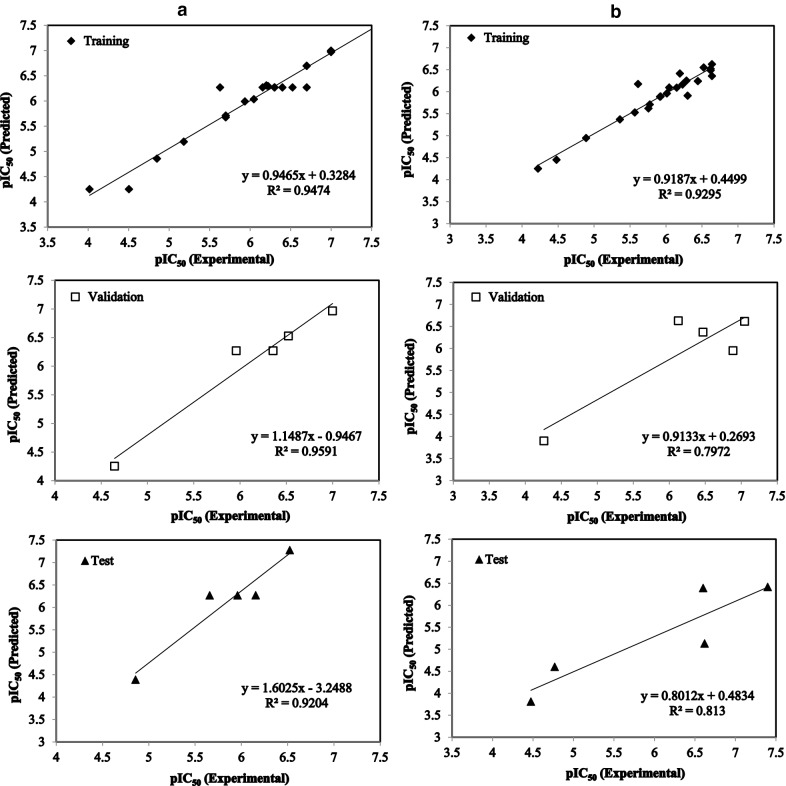
Plot of the pIC_50_ predicted (using artificial neural networks model) versus the experimental pIC_50_ for **a** 3D7 and **b** W2

The actual and predicted amounts of pIC_50_ of the used imidazolopiperazine derivatives as anti-malarial structures against 3D7 and W2 strain are represented in Table 6. There is good agreement between predicted and actual values of pIC_50_ in the proposed anti-malarial model for 3D7 activity and can be seen visually in Fig. 2. About model constructed for activity against W2 however the training and validation was acceptable but the prediction ability in the external test set was not as good as 3D7.

**Table 6:** Experimental and predicted activities (pIC_50_) of the imidazolopiperazine derivatives as anti-malarial structures against 3D7 and W2

Compound number	3D7			Compound number	W2		
	pIC_50_ (exp)	pIC_50_ (pred)	Residual		pIC_50_ (exp)	pIC_50_ (pred)	Residual
1	6.201	6.309	− 0.108	1	6.013	5.959	0.054
2	5.629	6.269	− 0.640	2	5.567	5.527	0.040
3	5.936	5.990	− 0.054	3	5.924	5.890	0.034
4	7	6.996	0.004	4^a^	4.258	3.901	0.357
5	5.699	5.711	− 0.012	5	5.757	5.620	0.137
6	6.699	6.695	0.004	6	6.638	6.624	0.014
7	6.155	6.269	− 0.114	7^a^	6.125	6.629	− 0.504
8	6.046	6.035	0.011	8	6.194	6.413	− 0.219
9	6.523	6.269	0.254	9	6.62	6.519	0.101
10	6.699	6.269	0.430	10^b^	6.602	6.389	0.213
11^b^	5.959	6.269	− 0.310	11	5.917	5.885	0.032
12^b^	6.155	6.269	− 0.114	12	6.229	6.164	0.065
13	5.699	5.677	0.022	13	5.775	5.709	0.066
14	5.18	5.192	− 0.012	14	5.36	5.369	− 0.009
15^a^	4.644	4.252	0.392	15^b^	4.769	4.600	0.169
16	4.503	4.252	0.251	16^b^	4.474	3.809	0.665
17^a^	7	6.965	0.035	17	6.523	6.550	− 0.027
18^b^	4.857	4.387	0.470	18	4.891	4.946	− 0.055
19	6.222	6.296	− 0.074	19	6.301	5.910	0.391
20	4.017	4.252	− 0.235	20	4.223	4.251	− 0.028
21	4.85	4.858	− 0.008	21	4.479	4.451	0.028
22	6.301	6.270	0.031	22^a^	6.469	6.369	0.100
23	6.301	6.269	0.032	23	6.149	6.091	0.058
24	7	6.971	0.029	24^a^	6.886	5.950	0.936
25	6.699	6.698	0.001	25^b^	6.62	5.130	1.490
26	7	6.987	0.013	26^a^	7.046	6.614	0.432
27^b^	6.523	7.278	− 0.755	27	6.62	6.485	0.135
28^a^	6.523	6.528	− 0.005	28	6.638	6.357	0.281
29^a^	6.357	6.269	0.088	29	6.444	6.240	0.204
30	7.523	7.526	− 0.003	30^b^	7.398	6.414	0.984
31	6.398	6.269	0.129	31	6.284	6.251	0.033
32^a^	5.959	6.268	− 0.309	32	6.046	6.095	− 0.049
33^b^	5.658	6.269	− 0.611	33	5.613	6.176	− 0.563

^a^The selected molecules as the validation data set

^b^The selected molecules as the test data set

Despite the good agreement between actual and predicted values in the two 3D7 and W2 models and specifically in the first one, but because the high number of descriptors (about 555 descriptors) which were selected in the variable selection step, there was the possibility of obtaining chancy models [22]. For evaluation of chance correlation, y-scrambling test was done. The dependent variable of the two 3D7 and W2 models (the experimental pIC_50_ of the selected derivatives) was randomly shuffled 30 times and ANN was run on them each time. The maximum correlation coefficient of the test set (R^2^_MP_) for these scrambled 3D7 and W2 models were 0.09 and 0.13 respectively. These low values of the correlation coefficients of the scrambled models (R^2^_MP_) in comparison to the original 3D7 and W2 models imply the absence of chance correlation.

### Applicability domain

Applicability domain (AD) of a QSAR model is an important point, because it defines the model limitations. Actually “the applicability domain of a (Q)SAR model is the response and chemical structure space in which the model makes predictions with a given reliability” [23].

**Fig. 3:**
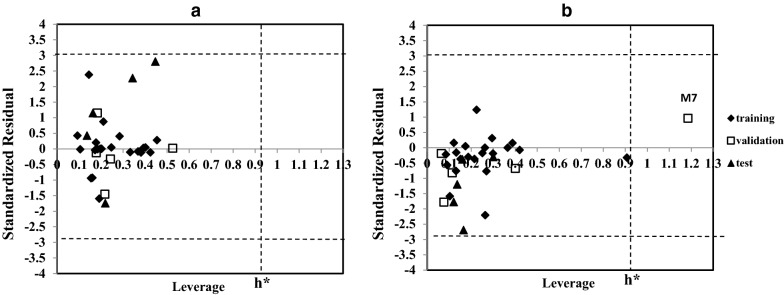
The Williams plots of the 33 compounds obtained by 6 descriptors used in artificial neural network models for **a** 3D7 and **b** W2

Different methods have been suggested for calculation of AD [24]. One of the recommended approaches to define AD is the method based on leverage and standard residual. The Williams plot that displays the standardized residuals versus leverage (hat diagonal) values is a way to verify the AD of a QSAR model [25, 26]. Leverage is proportional to the Mahalanobis distance of a query chemical from the centroid of the training set. For a given descriptor dataset **X**, the leverages are calculated with the (H = X (XʹX)^−1^Xʹ) equation, where Xʹ is the transpose of X matrix [24, 27]. The diagonal value (*h*_*i*_) represents the leverage value for *i*th point in the X dataset from the centre of the set of X observations. The higher leverage values represent the far compounds from the centre and they are more influential in model building [24]. It should be mentioned that a warning value for leverage is defined; so that if a query chemical has higher leverage than the warning value, it can be as unreliable prediction [24]. This warning leverage generally is equal to 3*p/n* where *p* is the number of model descriptors plus one (here *p *= 7), and *n* is the number of compounds used for the training model [24, 28]. It should be noted if the leverage of a query chemical was less than the warning value, there is not necessarily to be stayed on the range of the applicability domain of the model, and may be it has high standardized residuals. So in the Williams plot both of the two parameters (leverage values and standardized residuals) for surveying the AD of model has been considered. The Williams plots of 33 compounds of the models with 6 descriptors for 3D7 and W2 are displayed in Fig. 3.

## Discussion

In this research an artificial neural network was employed to gain a set of descriptors and to build a QSAR model for antimalarial activity. The controversial topic is how each step for QSAR model building such as data collection, model validation and prediction is performed.

The randomized selection of prediction and test subsets is a good method for external evaluation of the final model [29]. For each 3D7 and W2 model, As seen in Fig. 1, the two-dimensional PCA plot (PC1 vs. PC2) show that the molecules belongs to the three training, validation and test data sets are randomly distributed in the space of descriptors.

The other important step in any QSAR study is variable selection, because the method which is used for descriptor choosing has a great impact on all subsequent steps in drug design. The ideal path for variable selection is extensively search to all possible combinations of the initial descriptors, which is impossible except with small data set which have small number of descriptors [30].

For this purpose after using stepwise MLR (SMLR), the variables with the most significant Fisherʼs value (F) and with the highest correlation coefficient (R^2^) were selected. In this way variables with the most significant Fisherʼs value and the highest correlation coefficient were selected by inserting into/removing from the model. The number of 12 variables were checked using ANN method for training and validation sets. At the end it was found that the prediction ability of the models with 6 variables (reported in Table 2) are the best.

The next step was the evaluation of correlation in the selected descriptors. In the case of correlation between descriptors, the efficiency of the QSAR models are reduced and leads to biased estimation [31]. The pair correlation matrix was evaluated for the six descriptors of the two 3D7 and W2 models (Tables 3 and 4).It is clear from the Tables 3 and 4, no serious dependency is found in both descriptor set.

In addition to pair correlation, another kind of linear dependency can limit the accuracy of model which is known as multicollinearity which is shown the linear dependency of a variable (predictor) to all others [31]. Variance inflation factor (VIF), which is given in the following equation, is a popular diagnostic index for appearing multicollinearity [32].

$${\text{VIF}}_{\text{i}} = \frac{1}{{1 - R_{i}^{2} }}$$

where ${\text{R}}_{\text{i}}^{2}$ is the *R*^2^-value obtained by regressing the *i*th predictor on the other predictors [32].

As it is shown in the last column of Tables 3 and 4, all the calculated VIF are less than 3, and as regards to the proposed critical value for VIF that is equal to 5.0, the information of none of the six used descriptors for both 3D7 and W2 models has multicollinearity with the other descriptors and the resulting models are acceptable.

Looking at the results of model development and validation (Table 5), we find that the values of R^2^ and MSE of the training set for both of 3D7 and W2 models are good and express the good fitness of the models. Nevertheless it is necessary to use validation and test set to check the prediction ability of the anti-malarial models. As can be seen in Table 5, the statistics of validation and test sets of the model suggested for inhibitory against 3D7 strain were excellent (R^2^_val_ = 0.959, R^2^_test_ = 0.920) and the values of MSE for the two validation and test data sets were also good (0.051 and 0.254, respectively). The model generated for inhibitory against W2 strain with R^2^_val_ = 0.797 and R^2^_test_ = 0.813 was acceptable and the MSE values of its validation and test data sets (0.290 and 0.740, respectively) were not good in case of test set. It is clear from the results that the anti-3D7 activity model with the excellent statistics values for training, validation, and test (R^2^_train_ = 0.947, R^2^_val_ = 0.959, R^2^_test_ = 0.920) was better from the anti-W2 activity model and the latter was not very good but has acceptable performance which can be used for a brief estimation of activity against W2.

Also from the Williams plots (Fig. 3), it is clear that all the 33 compounds, except molecule No. 7 in W2 model have leverage values lower than the warning leverage. Also all the compounds were in the acceptable range of standardized residual (± 3σ). These results confirm that the prediction using six descriptors (which were selected by SMLR) in ANN models can be acceptable.

Also from the Williams plots (Fig. 3), it is clear that all the 33 compounds, except molecule No. 7 in W2 model have leverage values lower than the warning leverage. Also all the compounds were in the acceptable range of standardized residual (± 3σ). These results confirm that the prediction using six descriptors (which were selected by SMLR) in ANN models can be acceptable.

## Conclusion

Malaria is a deadly infectious disease, which is prevalent especially in the tropical developing countries. Resistance to existing anti-malarial drugs is a factor forcing researchers to develop or modify the anti-malarial compounds. The QSAR study with highlighting the structure activity relationships which correlate the compounds’ structural features with the observed anti-malarial activities could be a suitable way to design and to modify anti-malarial compounds. Actually in silico drug design methods, such as QSAR, play an important role in the drug design process due to saving money and time.

In this research, the anti-malarial activity of 33 imidazolopiperazine derivatives was investigated at 3D7 and W2 strain, using QSAR method. The linear methods, such as MLR and PLS models was not suitable but non-linear ANN showed good performance. The statistical parameters were used to evaluate the results. The results of R^2^, MSE and leverage value showed that the prediction ability of ANN method for estimation of the anti-malarial activity in imidazolopiperazine compounds is good and can be used as a virtual tool for synthesis of analogous compounds.

## Supplementary information

**Additional file 1.**Additional tables.

## Data Availability

All the information about datasets during and/or analysed during the current research are included in the manuscript, additional file and other required data is available from the corresponding author on reasonable request.

## Abbreviations

AD: applicability domain
ANN: artificial neural network
MLR: multiple linear regression
MSE: mean square error
PC: principal component
PCA: principal component analysis
PLS: partial least square
QSAR: quantitative structure activity relationship
SMLR: stepwise multiple linear regression
VIF: variance inflation factor

## References

[1] Mishra M, Mishra VK, Kashaw V, Iyer AK, Kashaw SK (2017). Comprehensive review on various strategies for antimalarial drug discovery. Eur J Med Chem.

[2] Biamonte MA, Wanner J, Le Roch KG (2013). Recent advances in malaria drug discovery. Bioorg Med Chem Lett.

[3] Marson BM, Vilhena R de O, Fachi MM, Pontes FLD, de Almeida BMM, Pontarolo R. Challenges and perspectives in malaria treatment. In: Malaria. Avid Science Publ; 2019. http://www.avidscience.com/book/malaria/.

[4] Flannery EL, Chatterjee AK, Winzeler EA (2013). Antimalarial drug discovery—approaches and progress towards new medicines. Nat Rev Microbiol.

[5] Calderón F, Wilson DM, Gamo F-J (2013). Antimalarial drug discovery: recent progress and future directions. Prog Med Chem.

[6] Ekins S, Mestres J, Testa B (2007). In silico pharmacology for drug discovery: applications to targets and beyond. Br J Pharmacol.

[7] Kumar Ojha P, Roy K (2015). The current status of antimalarial drug research with special reference to application of QSAR models. Comb Chem High Throughput Screen..

[8] Agrawal VK, Srivastava R, Khadikar PV (2001). QSAR Studies on some antimalarial sulfonamides. Bioorg Med Chem.

[9] Cheng F, Shen J, Luo X, Zhu W, Gu J, Ji R (2002). Molecular docking and 3-D-QSAR studies on the possible antimalarial mechanism of artemisinin analogues. Bioorg Med Chem.

[10] Katritzky AR, Kulshyn OV, Stoyanova-Slavova I, Dobchev DA, Kuanar M, Fara DC (2006). Antimalarial activity: a QSAR modeling using CODESSA PRO software. Bioorg Med Chem.

[11] Cardoso FJB, de Figueiredo AF, da Silva Lobato M, de Miranda RM, de Almeida RCO, Pinheiro JC (2008). A study on antimalarial artemisinin derivatives using MEP maps and multivariate QSAR. J Mol Model.

[12] Cheoymang A, Na-Bangchang K (2018). A systematic review: application of in silico models for antimalarial drug discovery. Afr J Pharm Pharmacol..

[13] Leong FJ, Zhao R, Zeng S, Magnusson B, Diagana TT, Pertel P (2014). A first-in-human randomized, double-blind, placebo-controlled, single- and multiple-ascending oral dose study of novel imidazolopiperazine KAF156 to assess its safety, tolerability, and pharmacokinetics in healthy adult volunteers. Antimicrob Agents Chemother.

[14] Chia PY, Hsu LY, Yeo TW (2018). Malaria in 2018: looking to the past and moving into the future. Ann Acad Med..

[15] Nagle A, Wu T, Kuhen K, Gagaring K, Borboa R, Francek C (2012). Imidazolopiperazines: lead optimization of the second-generation antimalarial agents. J Med Chem.

[16] Wu T, Nagle A, Kuhen K, Gagaring K, Borboa R, Francek C (2011). Imidazolopiperazines: hit to lead optimization of new antimalarial agents. J Med Chem.

[17] Todeschini R, Consonni V (2009). Molecular descriptors for chemoinformatics.

[18] Mauri A, Consonni V, Pavan M, Todeschini R (2006). Dragon software: an easy approach to molecular descriptor calculations. MATCH Commun Math Comput Chem..

[19] Yousefinejad S, Hemmateenejad B (2015). Chemometrics tools in QSAR/QSPR studies: a historical perspective. Chemom Intell Lab Syst.

[20] Yousefinejad S, Mahboubifar M, Rasekh S (2019). Prediction of different antibacterial activity in a new set of formyl hydroxyamino derivatives with potent action on peptide deformylase using structural information. Struct Chem.

[21] Hawkins DM (2004). The problem of overfitting. J Chem Inf Comput Sci.

[22] Gramatica P (2014). External evaluation of QSAR models, in addition to cross-validation: verification of predictive capability on totally new chemicals. Mol Inform..

[23] Netzeva TI, Worth AP, Aldenberg T, Benigni R, Cronin MD, Gramatica P (2005). Current status of methods for defining the applicability domain of (quantitative) structure–activity relationships. Altern Lab Anim.

[24] Sahigara F, Mansouri K, Ballabio D, Mauri A, Consonni V, Todeschini R (2012). Comparison of different approaches to define the applicability domain of QSAR models. Molecules.

[25] Gramatica P (2007). Principles of QSAR models validation: internal and external. QSAR Comb Sci.

[26] Yousefinejad S, Honarasa F, Montaseri H (2015). Linear solvent structure-polymer solubility and solvation energy relationships to study conductive polymer/carbon nanotube composite solutions. RSC Adv..

[27] Dimitrov S, Dimitrova G, Pavlov T, Dimitrova N, Patlewicz G, Niemela J (2005). A stepwise approach for defining the applicability domain of SAR and QSAR models. J Chem Inf Model.

[28] Honarasa F, Yousefinejad S, Nasr S, Nekoeina M (2015). Structure–electrochemistry relationship in non-aqueous solutions: predicting the reduction potential of anthraquinones derivatives in some organic solvents. J Mol Liq.

[29] Yousefinejad S, Eftekhari R, Honarasa F, Zamanian Z, Sedaghati F (2017). Comparison between the gas–liquid solubility of methanol and ethanol in different organic phases using structural properties of solvents. J Mol Liq.

[30] Yasri A, Hartsough D (2001). Toward an optimal procedure for variable selection and QSAR model building. J Chem Inf Comput Sci.

[31] Yoo W, Mayberry R, Bae S, Singh K, He QP, Lillard JW (2014). A study of effects of multicollinearity in the multivariable analysis. Int J Appl Sci Technol..

[32] Alin A (2010). Multicollinearity. Wiley Interdiscip Rev Comput Stat..

