# Supplementary material for: Quantitative structure–activity relationship to predict the anti-malarial activity in a set of new imidazolopiperazines based on artificial neural networks
Source: Malar J. 2019 Sep 14;18:310. doi: 10.1186/s12936-019-2941-5 (PMC6744662; doi:10.1186/s12936-019-2941-5)
Supplement: Supplementary file 1 — Additional file 1.Additional tables. [file 12936_2019_2941_MOESM1_ESM.pdf]

DOI: 10.1186/s12936-019-2941-5

Supporting information

Quantitative Structure-Activity Relationship to Predict the Antimalarial Activity in a Set of New Imidazolopiperazines Based on Artificial Neural Networks

Saeed Yousefinejad [a] *, Marjan Mahboubifar [b], Rayhaneh Eskandari [c]

[a] Research Center for Health Sciences, Institute of Health, Department of Occupational Health Engineering, School of Health, Shiraz University of Medical Sciences, Shiraz, Iran

[b] Medicinal and Natural Products Chemistry Research Center, Shiraz University of Medical Sciences, Shiraz, Iran

[c] Department of Chemistry, Shiraz Branch, Islamic Azad University, Shiraz, Iran

* Corresponding author:

yousefisa@sums.ac.ir; yousefinejad.s@gmail.com; Fax: +98 71 37256006;

Orcid ID: https://orcid.org/0000-0001-5940-1229 (S. Yousefinejad, Ph.D.)

DOI: 10.1186/s12936-019-2941-5

Table S1. Coeffitients, t and p-values of the six descriptors seleted by stepwise MLR which was used in 3D7 model.

Model		Unstandardized Coefficients		Standardized Coefficients	t	P-value
		B	Std. Error	Beta		
6	intercept	11.826	1.278		9.257	0.000
	GATS7m	-351.239	111.124	-0.514	-3.161	0.004
	Mor31u	2.656	0.606	0.505	4.380	0.000
	Mor06u	0.378	0.095	0.461	3.984	0.000
	R2p+	27.696	7.793	0.414	3.554	0.001
	GATS4m	-288.159	131.318	-0.340	-2.194	0.037
	R3e+	-6.042	2.760	-0.261	-2.189	0.038

Table S2. Model sumarry of the first six models containted 1 to 6 descriptors seleted by stepwise MLR which was used in 3D7 model.

Model	R	R Square	Adjusted R Square	Std. Error of the Estimate	Include descriptors
1	0.576	0.332	0.311	0.67025	GATS7m
2	0.698	0.487	0.453	0.59726	GATS7m,Mor31u
3	0.782	0.611	0.571	0.52910	GATS7m, Mor31u,Mor06u
4	0.817	0.668	0.621	0.49733	GATS7m, Mor31u, Mor06u, R2p+
5	0.845	0.714	0.661	0.47040	GATS7m, Mor31u, Mor06u, R2p+, GATS4m
6	0.871	0.758	0.702	0.44049	GATS7m, Mor31u, Mor06u, R2p+, GATS4m, R3e+

Table S3. ANOVA results for MLR model contained first six descriptors used in 3D7 model.

Model		Sum of Squares	F	p-value
6	Regression	6.932	15.430	0.000
	Residual	13.926		
	Total	20.858		

DOI: 10.1186/s12936-019-2941-5

Table S4. Coeffitients, t and p-values of the six descriptors seleted by stepwise MLR which was used in W2 model.

Model		Unstandardized Coefficients		Standardized Coefficients	t	p-value
		B	Std. Error	Beta		
8	intercept	-44.268	18.923		-2.339	0.027
	Mor20p	0.678	0.085	0.928	7.941	0.000
	MATS7m	88.680	15.337	0.628	5.782	0.000
	RDF020m	2.556	0.387	0.718	6.600	0.000
	MLOGP	0.760	0.170	0.515	4.475	0.000
	BEHm3	-12.976	3.430	-0.421	-3.783	0.001
	Mor23u	-0.936	0.304	-0.360	-3.080	0.005

Table S5. Model sumarry of the first six models containted 1 to 6 descriptors seleted by stepwise MLR which was used in W2 model.

Model	R	R Square	Adjusted R Square	Std. Error of the Estimate	Include descriptor
1	0.525	0.275	0.252	0.71008	H3m,
2	0.624	0.390	0.349	0.66232	H3m, Mor20p,
3	0.713	0.509	0.458	0.60431	H3m, Mor20p, MATS7m
4	0.789	0.622	0.568	0.53928	H3m, Mor20p, MATS7m, RDF020m,
5	0.770	0.592	0.550	0.55063	Mor20p, MATS7m, RDF020m,
6	0.827	0.685	0.640	0.49273	Mor20p, MATS7m, RDF020m, MLOGP
7	0.862	0.743	0.695	0.45349	Mor20p, MATS7m, RDF020m, MLOGP, BEHm3
8	0.901	0.811	0.768	0.39558	Mor20p, MATS7m, RDF020m, MLOGP, BEHm3, Mor23u

Table S6. ANOVA results for MLR model contained first six descriptors used in W2 model.

Model		Sum of Squares	F	p-value
8	Regression	17.496	18.635	0.000
	Residual	4.068		
	Total	21.564		

DOI: 10.1186/s12936-019-2941-5

Table S1. The name and numerical values of selected variables for 3D7 model.

compound number	GATS7m	Mor31u	Mor06u	R2p+	GATS4m	R3e+
1	0.009	0.056	-2.557	0.101	0.011	0.144
2	0.009	0.123	-4.683	0.087	0.01	0.129
3	0.011	0.27	-4.041	0.098	0.011	0.143
4	0.007	-0.063	-2.255	0.089	0.008	0.202
5	0.009	-0.026	-2.247	0.103	0.01	0.252
6	0.008	0.059	-2.2	0.094	0.01	0.22
7	0.009	0	-1.44	0.077	0.011	0.164
8	0.009	-0.107	-2.918	0.089	0.011	0.203
9	0.009	0.229	-1.551	0.081	0.011	0.182
10	0.009	-0.101	-1.904	0.089	0.011	0.206
11	0.009	0.072	-3.291	0.075	0.01	0.164
12	0.009	0.179	-3.172	0.083	0.01	0.168
13	0.01	0.04	-2.615	0.088	0.011	0.203
14	0.009	-0.04	-2.015	0.1	0.012	0.233
15	0.009	-0.157	-3.13	0.097	0.011	0.227
16	0.01	-0.067	-3.116	0.098	0.011	0.227
17	0.009	0.019	-2.402	0.097	0.01	0.23
18	0.01	-0.184	-2.149	0.087	0.011	0.199
19	0.01	0.074	-1.264	0.097	0.011	0.187
20	0.013	0.28	-3.634	0.083	0.013	0.167
21	0.01	-0.267	-2.378	0.084	0.011	0.174
22	0.008	-0.092	-2.305	0.092	0.01	0.232
23	0.01	-0.07	-0.735	0.087	0.01	0.201
24	0.01	0.098	-2.518	0.104	0.011	0.152
25	0.01	0.354	-1.482	0.096	0.012	0.185
26	0.009	-0.107	-1.947	0.13	0.012	0.194
27	0.009	-0.124	-0.386	0.115	0.012	0.187
28	0.007	-0.032	-1.888	0.093	0.01	0.277
29	0.008	-0.125	-1.491	0.084	0.01	0.244
30	0.007	-0.074	-1.58	0.123	0.011	0.253
31	0.008	-0.154	-0.12	0.087	0.011	0.181
32	0.009	-0.043	-3.304	0.081	0.01	0.192
33	0.008	-0.32	-1.868	0.088	0.009	0.213

DOI: 10.1186/s12936-019-2941-5

Table S2. The name and numerical values of selected variables for W2 model.

compound number	Mor20p	MATS7m	RDF020m	MLOGP	BEHm3	Mor23u
1	1.053	0.997	0.978	1.275	3.67	-1.224
2	1.591	0.995	1.041	2.514	3.781	-1.652
3	1.159	0.988	0.983	2.127	3.688	-1.438
4	1.015	1.008	0.403	3.006	3.684	-0.747
5	1.244	1.008	0.646	3.155	3.659	-0.957
6	1.091	1.003	0.779	2.377	3.688	-0.946
7	0.745	1.007	1.636	2.595	3.69	-0.687
8	1.12	1.004	0.75	2.595	3.694	-1.017
9	1.277	1.014	0.75	3.021	3.704	-0.838
10	1.321	1.004	0.685	2.81	3.707	-1.393
11	1.56	0.999	0.802	3.615	3.753	-1.296
12	1.764	0.999	0.83	3.615	3.753	-1.911
13	1.393	1	0.697	2.435	3.692	-0.817
14	1.228	1.005	0.777	2.486	3.734	-1.103
15	1.307	0.993	0.976	1.727	3.695	-1.399
16	1.278	0.992	0.812	2.218	3.694	-1.063
17	1.266	1.001	0.777	2.377	3.687	-1.343
18	1.346	0.997	0.7	2.81	3.706	-1.023
19	1.233	0.999	0.705	3.021	3.721	-0.978
20	0.471	0.992	0.828	2.4	3.634	-1.019
21	1.06	1.002	0.762	1.191	3.702	-0.968
22	1.157	1	0.771	2.377	3.688	-1.039
23	1.308	1.003	0.685	2.81	3.707	-1.454
24	1.217	0.996	0.812	2.218	3.689	-1.19
25	1.368	0.997	0.715	2.648	3.707	-1.516
26	1.268	1.006	0.776	2.486	3.692	-1.224
27	1.247	1.007	0.684	2.916	3.709	-1.528
28	1.258	1.005	0.764	2.754	3.689	-1.25
29	1.401	1.006	0.675	3.184	3.708	-0.903
30	1.246	1.008	0.768	2.863	3.692	-1.11
31	0.809	1.009	1.54	3.289	3.709	-0.27
32	0.656	1.003	0.809	3.184	3.708	-0.861
33	1.073	1.004	0.653	3.184	3.708	-1.157
